# A Shape Reconstruction and Measurement Method for Spherical Hedges Using Binocular Vision

**DOI:** 10.3389/fpls.2022.849821

**Published:** 2022-05-04

**Authors:** Yawei Zhang, Jin Gu, Tao Rao, Hanrong Lai, Bin Zhang, Jianfei Zhang, Yanxin Yin

**Affiliations:** ^1^College of Engineering, China Agricultural University, Beijing, China; ^2^Research Center of Intelligent Equipment, Beijing Academy of Agriculture and Forestry Sciences, Beijing, China; ^3^National Research Center of Intelligent Equipment for Agriculture, Beijing, China; ^4^Nanjing Institute of Agricultural Mechanization, Ministry of Agriculture and Rural Affairs, Nanjing, China

**Keywords:** spherical hedges, shape reconstruction, binocular vision, dimension measurement, 3D point cloud

## Abstract

The center coordinate and radius of the spherical hedges are the basic phenotypic features for automatic pruning. A binocular vision-based shape reconstruction and measurement system for front-end vision information gaining are built in this paper. Parallel binocular cameras are used as the detectors. The 2D coordinate sequence of target spherical hedges is obtained by region segmentation and object extraction process. Then, a stereo correcting algorithm is conducted to keep two cameras to be parallel. Also, an improved semi-global block matching (SGBM) algorithm is studied to get a disparity map. According to the disparity map and parallel structure of the binocular vision system, the 3D point cloud of the target is obtained. Based on this, the center coordinate and radius of the spherical hedges can be measured. Laboratory and outdoor tests on shape reconstruction and measurement are conducted. In the detection range of 2,000–2,600 mm, laboratory test shows that the average error and average relative error of standard spherical hedges radius are 1.58 mm and 0.53%, respectively; the average location deviation of the center coordinate of spherical hedges is 15.92 mm. The outdoor test shows that the average error and average relative error of spherical hedges radius by the proposed system are 4.02 mm and 0.44%, respectively; the average location deviation of the center coordinate of spherical hedges is 18.29 mm. This study provides important technical support for phenotypic feature detection in the study of automatic trimming.

## Introduction

With the vigorous development of urban greening, trimming or pruning hedges to desired shape regulars is one of the major tasks in urban plant landscape construction. Manual trimming using large scissors or power tools causes a significant load on the person executing this task. The semi-automated trimmer, however, also needs a driver operating, consumes most time, and is difficult to control working accuracy. Therefore, the development of automatic and intelligent pruning robots has drawn increasing attention.

To automatically trim hedges, finding the basic phenotypic information of hedges is the key. In a complex outdoor environment, an adaptive hedge horizontal cross-section center detection algorithm was proposed to obtain the hedge’s horizontal cross-section center in real time by inputting the top view image of the hedge. This detection algorithm could be truly applied in the vehicle-mounted system (Li et al., 2022). A TrimBot2020 robotic platform equipped with a pentagon-shaped rig of five pairs of stereo cameras was developed for navigation and 3D reconstruction, which can build the model of bush or hedges and be used as the input for the trimming operation (Strisciuglio et al., 2018). An arm-mounted vision approach was studied to scan a specified shape and fit it into the reconstructed point cloud, and then, a co-mounted trimming tool could cut the bush using an automatically planned trajectory, which ensured flexibility *via* a vision-based shape fitting module that allows fitting an arbitrary mesh into a bush at hand (Kaljaca et al., 2019a,b). Besides, the binocular vision system has great application in picking robots for object recognition and orientation. A litchi-picking robot based on binocular vision was developed to identify and locate the target and then provide information for collision-free motion planning. The results show that the success rate of path determination is 100% for the laboratory’s picking scene (Ye et al., 2021). Herein, vision sensing technology was widely used in characteristic recognition of fruits and vegetables and movement navigation of picking robots, such as tomatoes, apples, and Hangzhou White Chrysanthemums (Ji et al., 2017; Lili et al., 2017; Yang et al., 2018; Jin et al., 2020). From the above research, it can be concluded that binocular stereo vision technology has been widely used in agricultural robotics for three-dimensional (3D) reconstruction, measurement, navigation, etc. As the “eye” of the pruning robot, the shape reconstruction and dimension measurement of target objects provide a crucial information for the follow-up operation.

In this paper, a parallel binocular vision is constructed to complete the 3D reconstruction of spherical hedges, and high accuracy is achieved in both spherical center positioning and radius measurement. The 3D reconstruction contains two-dimensional (2D) image extraction, binocular camera calibration, stereo correcting, stereo matching, and sharp reconstruction. Herein, in this paper, stereo matching is a key technology of shape reconstruction, and an improved semi-global block matching (SGBM) algorithm was proposed in this study to get a good disparity map. Based on this, the center coordinate of spherical hedges and their radius is finally realized by processing the point cloud data.

## Materials and Methods

### Description of the Measurement System

**FIGURE 1:**
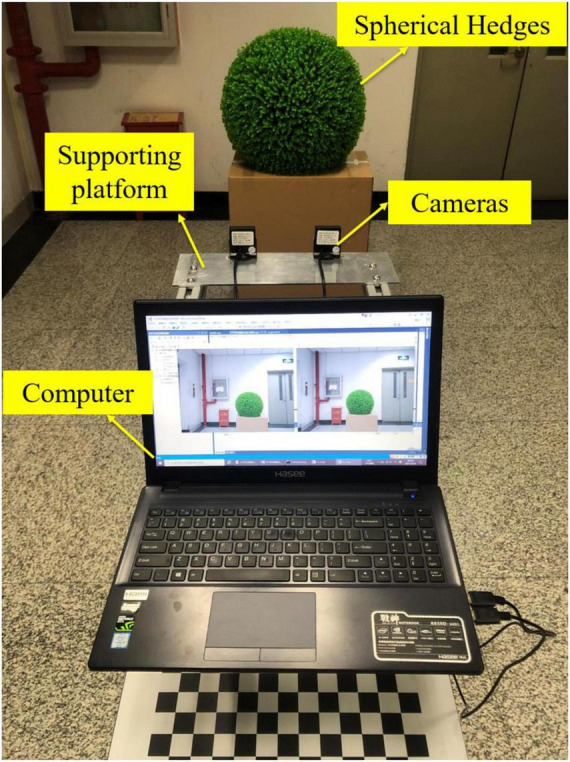
Schematic diagram of the binocular vision system.

To obtain point cloud information and reshape spherical hedges, a binocular vision system is used for measurement. The binocular vision system consists of two RMONCAM G200 cameras and a supporting platform. The cameras are mounted on the slider, and the positions of the cameras can be moved on the slider rail. The distance between two cameras can be set to 80, 100, 120, 140, and 160 mm. All experiments are involved in this paper, and the distance between the two cameras is set to 140 mm. The shape reconstruction and measurement system are programmed using Microsoft Visual Studio 2015, OpenCV3.4.10, and MATLAB2018a. The focus length, maximum frame rate, pixel size, and image resolution of a utilized camera are 2.8 mm, 60 fps, 3.0 μm × 3.0 μm, and 1,920 × 1,200 pixels, respectively. Figure 1 shows the schematic diagram of the binocular vision system.

**FIGURE 2:**
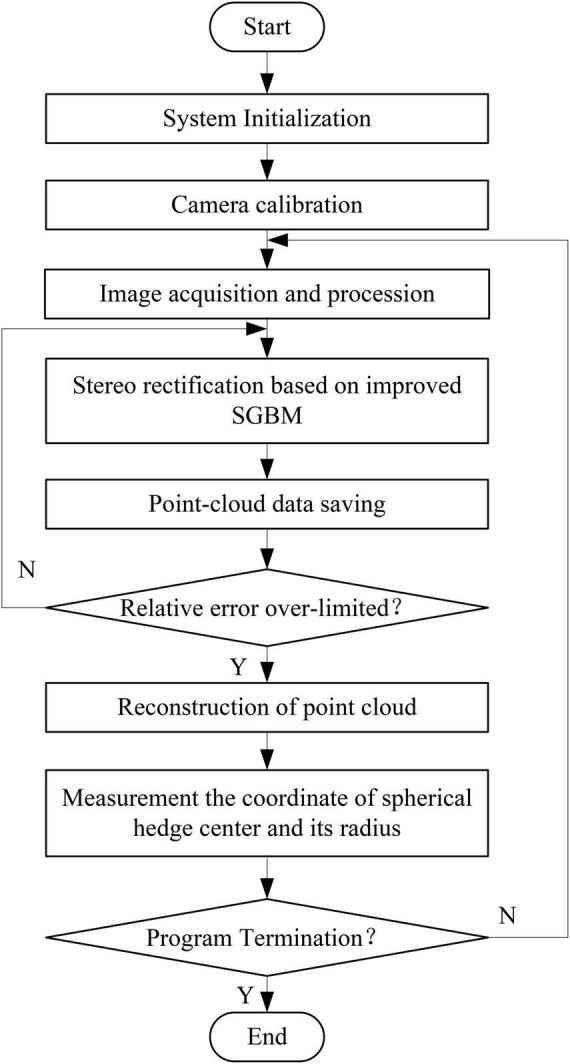
Flow chart of the measurement system.

When conducting experiments, the spherical hedges are placed in front of the cameras. Then, the system captures the current images. Next, the images are transmitted to the computer. Afterward, image processing is called to obtain the point cloud data of spherical hedges. Based on this, the shape reconstruction graph is obtained. Finally, the radius and center coordinate of spherical hedges are calculated. Figure 2 shows the flowchart of the measurement system.

### Camera Calibration and Image Processing

#### Monocular Vision Calibration

**FIGURE 3:**
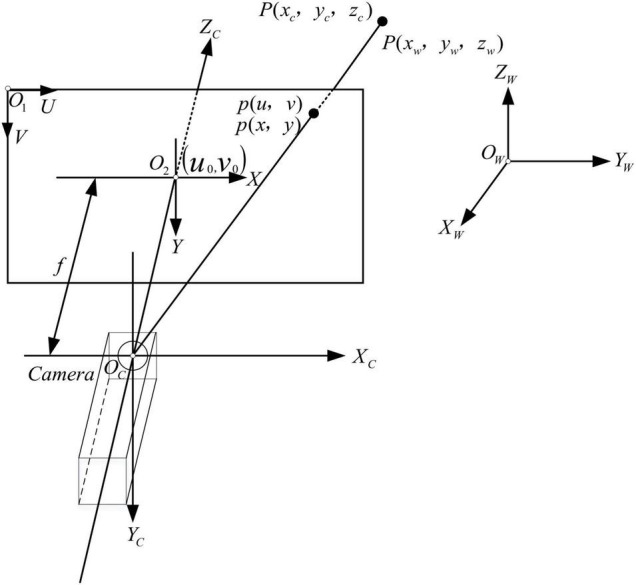
Schematic diagram of pinhole imaging.

Camera calibration is an important task because it directly determines the accuracy of 3D reconstruction (Long and Dongri, 2019). According to Zhang’s camera plane calibration method, the calibration test of a monocular camera is carried out first. Figure 3 presents the schematic diagram of pinhole imaging, *O*_*C*_−*X*_*C*_*Y*_*C*_*Z*_*C*_ is the camera coordinate system and *O*_*W*_−*X*_*W*_*Y*_*W*_*Z*_*W*_ is the world coordinate system; *O*_1_−*UV*is the pixel coordinate system and *O*_2_−*XY* is the image coordinate system. *P*(*x*_*w*_, *y*_*w*_, *z*_*w*_) is the world coordinate of point *P*, and its corresponding camera coordinate in camera is *P*(*x*_*c*_, *y*_*c*_, *z*_*c*_) and its pixel coordinate is *p*(*u*, *v*).

Converting from world coordinate system to pixel coordinate system needs to follow several transformations: transformation between world coordinate system and camera coordinate system; transformation between camera coordinate system and image coordinate system; and transformation between image coordinate system and pixel coordinate system.

The transformation between pixel coordinate system and image coordinate system is expressed as


(1) $$\left(\begin{array}{c} u\\ v\\ 1 \end{array}\right)=\left(\begin{array}{ccc} s_{x} & 0 & u_{0}\\ 0 & s_{y} & v_{0}\\ 0 & 0 & 1 \end{array}\right)\left(\begin{array}{c} x\\ y\\ 1 \end{array}\right)$$

where *s*_*x*_ is the pixel size of 1 mm in the *x*-direction of *O*_2_−*XY* and *s*_*y*_ is the pixel size of 1 mm in the *y*-direction of *O*_2_−*XY*.

The transformation between the camera coordinate system and image coordinate can be obtained from the pinhole imaging theory. It is formularized as


(2) $$z_{c}\left(\begin{array}{c} x\\ y\\ 1 \end{array}\right)=\left(\begin{array}{cccc} f & 0 & 0 & 0\\ 0 & f & 0 & 0\\ 0 & 0 & 1 & 0 \end{array}\right)\left(\begin{array}{c} x_{c}\\ y_{c}\\ z_{c}\\ 1 \end{array}\right)$$

where *f* is the focal length of the camera.

The transformation between the camera coordinate system and the world coordinate system can be obtained through rotation and translation. The transformation relationships are expressed as


(3) $$\left(\begin{array}{c} x_{c}\\ y_{c}\\ z_{c}\\ 1 \end{array}\right)=\left(\begin{array}{cc} R & T\\ 0^{T} & 1 \end{array}\right)\left(\begin{array}{c} x_{w}\\ y_{w}\\ z_{w}\\ 1 \end{array}\right)$$

where *R* and *T* represent the rotation matrix and the horizontal movable matrix.

Herein, the transformation between world coordinate system to pixel coordinate system can be determined by


(4) $$\begin{array}{c} z_{c}\left(\begin{array}{c} u\\ v\\ 1 \end{array}\right)=\left(\begin{array}{ccc} s_{x} & 0 & u_{0}\\ 0 & s_{y} & v_{0}\\ 0 & 0 & 1 \end{array}\right)\left(\begin{array}{cccc} f & 0 & 0 & 0\\ 0 & f & 0 & 0\\ 0 & 0 & 1 & 0 \end{array}\right)\left(\begin{array}{c} x_{c}\\ y_{c}\\ z_{c}\\ 1 \end{array}\right)=M_{1}M_{2}\left(\begin{array}{c} x_{w}\\ y{}_{w}\\ z_{w}\\ 1 \end{array}\right)\\ =M\left(\begin{array}{c} x_{w}\\ y{}_{w}\\ z_{w}\\ 1 \end{array}\right) \end{array}$$

where $M_{1}=\left(\begin{array}{cccc} f_{x} & 0 & u_{0} & 0\\ 0 & f_{y} & v_{0} & 0\\ 0 & 0 & 1 & 0 \end{array}\right)$, $M_{2}=\left(\begin{array}{cc} R & T\\ 0^{T} & 1 \end{array}\right)$, *M* = *M*_1_ • *M*_2_, *f*_*x*_ = *f* • *s*_*x*_, *f*_*y*_ = *f* • *s*_*y*_. The *f*_*x*_, *f*_*y*_, *u*_0_, and *v*_0_ are camera intrinsic parameters, and thus, *M*_1_ represents the camera’s intrinsic parameter matrix. The *M*_2_ represents the camera’s extrinsic parameter matrix; hence, *M* represents the projection matrix of the camera.

Moreover, a high-order polynomial model is adopted to correct the image distortion. The high-order polynomial model is expressed as


(5) $$\left[\begin{array}{c} x_{c}-x_{0}\\ y_{c}-y_{0} \end{array}\right]=L\left(r\right)\left[\begin{array}{c} x-x_{0}\\ y-y_{0} \end{array}\right]$$

where *L*(*r*) = 1 + *k*_1_*r* + *k*_2_*r*^2^ + *k*_3_*r*^3^ + …, $r=\sqrt{{\left(x-x_{0}\right)}^{2}+{\left(y-y_{0}\right)}^{2}}$, *x* and *y* refer to the horizontal and vertical coordinate values before correction, respectively, *x*_*c*_ and *y*_*c*_ refer to the horizontal and vertical coordinate values after correction, respectively, *x*_0_ and *y*_0_ refer to coordinate values of the center of the distorted image. Herein, a polynomial distortion correction model of the camera can be expressed as


(6) $$\left\{ \begin{array}{c} x_{c}=x\left(1+k_{1}r^{2}+k_{2}r^{4}+k_{3}r^{6}+\ldots \right)+2p_{1}xy+p_{2}\left(r^{2}+2x^{2}\right)\\ y_{c}=y\left(1+k_{1}r^{2}+k_{2}r^{4}+k_{3}r^{6}+\ldots \right)+2p_{2}xy+p_{1}\left(r^{2}+2y^{2}\right) \end{array}\right.$$

where *k*_1_, *k*_2_, and *k*_3_ are radial distortion coefficients, *p*_1_ and *p*_2_ are tangential distortion coefficients. Herein, *k*_1_, *k*_2_, *k*_3_, *p*_1_, and *p*_2_ are also camera intrinsic parameters.

Herein, the camera calibration toolbox (Toolbox_Calib) in MATLAB is used for monocular vision calibration. The calibration process of a monocular vision camera is as follows: image calibration, calibration chessboard extraction, corner points extraction, intrinsic and extrinsic parameters calculation, and calibration error analysis.

#### Binocular Vision Calibration

**FIGURE 4:**
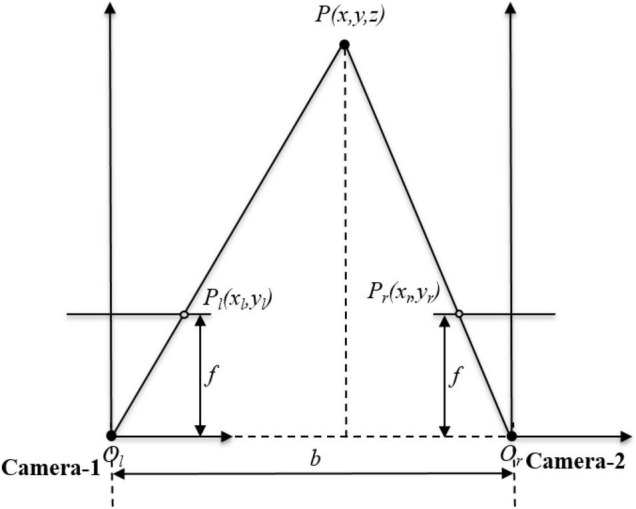
The parallel structure of the binocular vision system.

The binocular vision calibration is conducted based on the monocular vision calibration; through calibration test, the intrinsic matrix and extrinsic matrix of a camera can be obtained. In this paper, a parallel binocular stereo vision system is built. The two cameras are the same and mounted at the same height, and its front end is parallel and level. The parallel structure of the binocular vision system is shown in Figure 4. The left camera is called the Camera-1 and the left camera is called the Camera-2. Set the camera coordinate of Camera-1 as the reference world coordinate system. As indicated above, *P*(*x*_*w*_, *y*_*w*_, *z*_*w*_)is the world coordinate of point *P*. Its corresponding image coordinate in Camera-1 is *p*_*l*_(*x*_*l*_, *y*_*l*_) and its corresponding image coordinate in Camera-2 is *p*_*r*_(*x*_*r*_, *y*_*r*_).

According to the principle of similar triangles, it can be obtained as


(7) $$\left\{\begin{array}{c} x_{w}\\ y_{w}\\ z_{w} \end{array}\right\}=\left\{\begin{array}{c} \frac{z}{f}x_{l}\\ \frac{z}{f}y_{l}\\ \frac{bf}{x_{l}-x_{r}} \end{array}\right\}$$

where *b* is the baseline distance of Camera-1 and Camera-2, *f* is the focal length of the camera, and *x*_*l*_−*x*_*r*_ is the disparity value.

As in Equation 3, the transformation between the camera coordinate system of Camera-1 and world coordinate can be obtained through rotating vector *R*_*l*_ and translation vector *T*_*l*_, and the transformation between camera coordinate system of Camera-2 and world coordinate can be obtained through rotating vector *R*_*r*_ and translation vector *T*_*r*_. Therefore, the transformation between camera coordinate systems of Camera-1 and Camera-2 can be represented as


(8) $$\left\{ \begin{array}{c} R=R_{l}R_{r}^{-1}\\ T=T_{l}-R_{l}R_{r}^{-1}T_{r} \end{array}\right.$$

The pixel coordinates of point *P* in Camera-1 and Camera-2 are *p*_*l*_(*u*_*l*_, *v*_*l*_) and *p*_*r*_(*u*_*r*_, *v*_*r*_), respectively. According to Equation 4, the transformation between world coordinate system to pixel coordinate system can be represented as


(9) $$\begin{array}{c} z_{cl}\left(\begin{array}{c} u_{l}\\ v_{l}\\ 1 \end{array}\right)=\left(\begin{array}{cccc} f_{xl} & 0 & u_{0l} & 0\\ 0 & f_{yl} & v_{0l} & 0\\ 0 & 0 & 1 & 0 \end{array}\right)\left(\begin{array}{cc} R_{l} & T_{l}\\ 0 & 1 \end{array}\right)\left(\begin{array}{c} x_{w}\\ y{}_{w}\\ z_{w}\\ 1 \end{array}\right)\\ =\left(\begin{array}{cccc} a_{11}^{1} & a_{12}^{1} & a_{13}^{1} & a_{14}^{1}\\ a_{21}^{1} & a_{22}^{1} & a_{23}^{1} & a_{24}^{1}\\ a_{31}^{1} & a_{32}^{1} & a_{33}^{1} & a_{34}^{1} \end{array}\right)\left(\begin{array}{c} x_{w}\\ y{}_{w}\\ z_{w}\\ 1 \end{array}\right) \end{array}$$

(10) $$\begin{array}{c} z_{cr}\left(\begin{array}{c} u_{r}\\ v_{r}\\ 1 \end{array}\right)=\left(\begin{array}{cccc} f_{xr} & 0 & u_{0r} & 0\\ 0 & f_{yr} & v_{0r} & 0\\ 0 & 0 & 1 & 0 \end{array}\right)\left(\begin{array}{cc} R_{r} & T_{r}\\ 0 & 1 \end{array}\right)\left(\begin{array}{c} x_{w}\\ y{}_{w}\\ z_{w}\\ 1 \end{array}\right)\\ =\left(\begin{array}{cccc} a_{11}^{2} & a_{12}^{2} & a_{13}^{2} & a_{14}^{2}\\ a_{21}^{2} & a_{22}^{2} & a_{23}^{2} & a_{24}^{2}\\ a_{31}^{2} & a_{32}^{2} & a_{33}^{2} & a_{34}^{2} \end{array}\right)\left(\begin{array}{c} x_{w}\\ y{}_{w}\\ z_{w}\\ 1 \end{array}\right) \end{array}$$

To solve the world coordinate [*x*_*w*_, *y*_*w*_, *z*_*w*_]^*T*^ of point *P*, taking the optical central position of Camera-1 as origin, an inhomogeneous linear equation is obtained through getting rid of *Z*_cl_ and *Z*_cr_ in Equations 9, 10.


(11) $$\left\{ \begin{array}{c} \left(u_{l}a_{31}^{1}-a_{11}^{1}\right)x_{W}+\left(u_{l}a_{32}^{1}-a_{12}^{1}\right)y_{W}+\left(u_{l}a_{33}^{1}-a_{13}^{1}\right)z_{W}=a_{14}^{1}-u_{l}a_{34}^{1}\\ \begin{array}{c} \left(v_{l}a_{31}^{1}-a_{21}^{1}\right)x_{W}+\left(v_{l}a_{32}^{1}-a_{22}^{1}\right)y_{W}+\left(v_{l}a_{33}^{1}-a_{23}^{1}\right)z_{W}=a_{24}^{1}-v_{l}a_{34}^{1}\\ \begin{array}{c} \left(u_{\text{r}}a_{31}^{2}-a_{11}^{2}\right)x_{W}+\left(u_{r}a_{32}^{2}-a_{12}^{2}\right)y_{W}+\left(u_{r}a_{33}^{2}-a_{13}^{2}\right)z_{W}=a_{14}^{2}-u_{r}a_{34}^{2}\\ \left(v_{\text{r}}a_{31}^{2}-a_{21}^{2}\right)x_{W}+\left(v_{r}a_{32}^{2}-a_{22}^{2}\right)y_{W}+\left(v_{r}a_{33}^{2}-a_{23}^{2}\right)z_{W}=a_{24}^{2}-v_{r}a_{34}^{2} \end{array} \end{array} \end{array}\right.$$

Up to now, for one point in space, as long as we obtain its pixel coordinates in Camera-1 and Camera-2, its world coordinates can be solved by Equation 11.

#### Region Segmentation and Object Extraction

**FIGURE 5:**
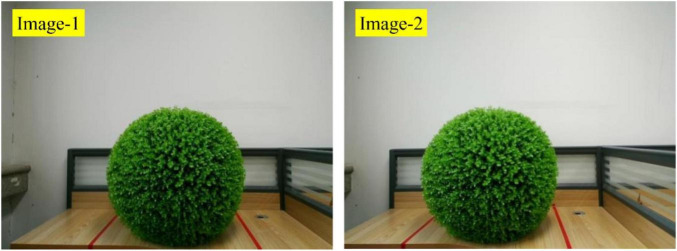
The Image-1 and Image-2.

After calibration, the binocular vision system can be used to capture images. The images captured by Camera-1 and Camera-2 are called Image-1 and Image-2, respectively. Figure 5 shows Image-1 and Image-2.

**FIGURE 6:**
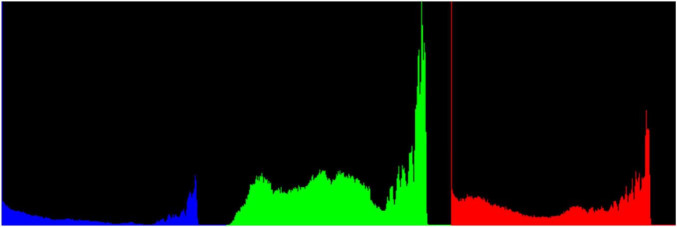
RGB color histogram of Image-2.

Take Image-2 as an example to introduce the hedges extraction process. The RGB color histogram of Image-2 is shown in Figure 6, which shows that green color accounts for the largest proportion. Ultra-green extraction of green plant images has a good effect on distinguishing the green plants from the surrounding environment, and it is the most commonly used grayscale method for crop recognition or weed recognition. The excess green index (*ExG*) of ultra-green algorithm is set to *E*x*G* = 2*G*−*R*−*B*.

**FIGURE 7:**
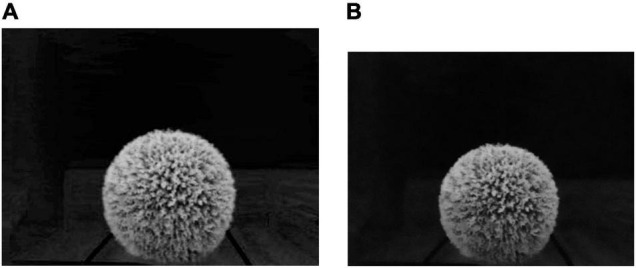
**(A)** The gray image of Image-2 obtained after the Ultra-green algorithm. **(B)** The bilateral filtered image of Image-2.

Figure 7A is the 2G-R-B gray image of Image-2. The bilateral filtering for image denoising is used for image noise removal. Figure 7B is the bilateral filtered image of Image-2, which shows that the image boundary features can be most reserved.

Then, gamma correction was studied to enhance the contrast between the target hedges and the surrounding environment under strong light and weak light. The gamma formula can be expressed as


(12) $$y={\left(x+esp\right)}^{\gamma }$$

where, *x* ∈ [0,1], *y* ∈ [0,1], *esp* is the compensation factor, and γ is the gamma coefficient.

**FIGURE 8:**
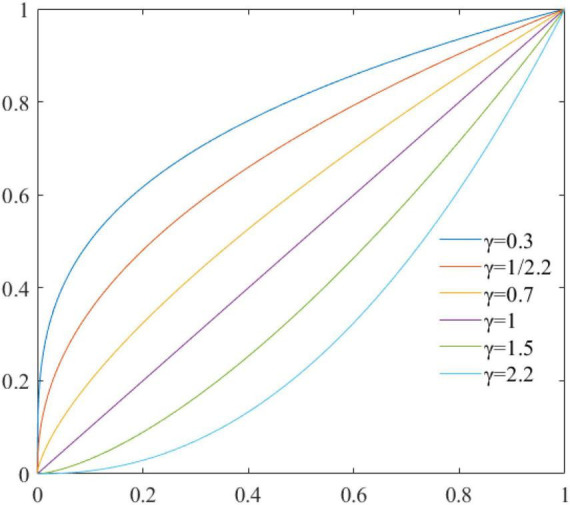
The gamma transform graph at different γ values.

Figure 8 shows the grayscale mapping relationship between the output image and the input image with different γ values.

**FIGURE 9:**
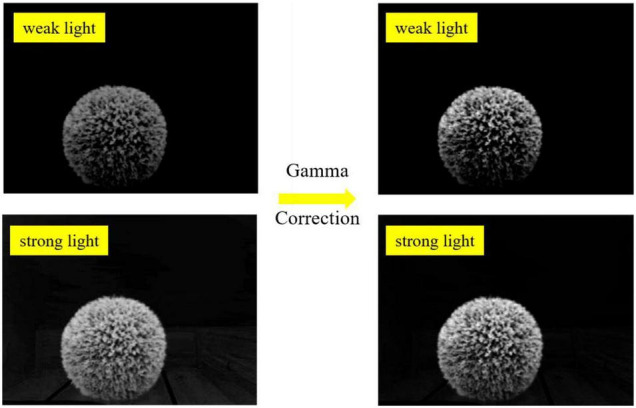
The enhanced contrast results of spherical hedge images in weak light and strong light.

From Figure 8, it can be seen that different γ values should be used when performing gamma transformations for images with different grayscale distributions. In this paper, the contrast has been enhanced to some extent after gamma correction as shown in Figure 9, when γequals 1.5.

**FIGURE 10:**
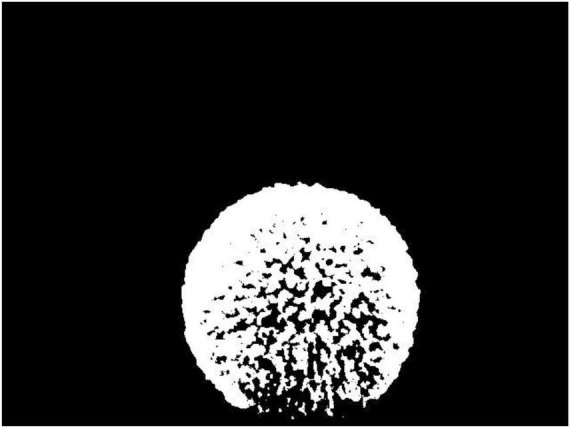
The Image-2 after binarization.

At last, the image binarization best treatment threshold is obtained using the maximum between-class variance method (OTSU), hereafter, the 2D coordinate sequence of spherical hedges can be obtained from Image-2 (Caraffa et al., 2015). Figure 10 shows the binary image of Image-2.

### Shape Reconstruction and Measurement

#### Stereo Image Rectification

**FIGURE 11:**
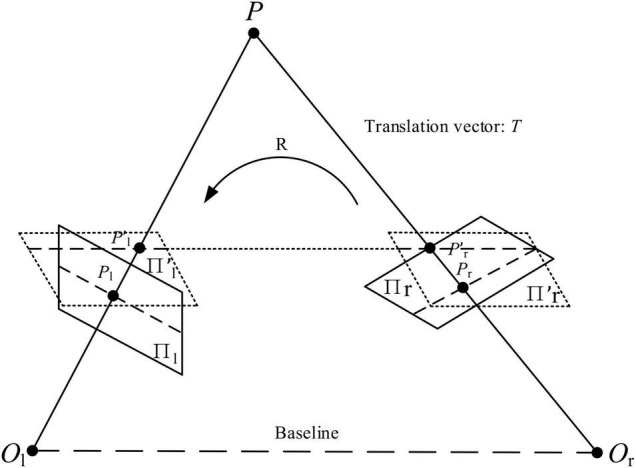
Schematic diagram of Bouguet’s algorithm.

It is difficult to align the two cameras in this binocular vision system to be perfectly parallel (Wu et al., 2017). After binocular vision calibration, the stereo image rectification is used based on Bouguet’s algorithm to ensure that the cameras are completely parallel. Figure 11 shows the algorithmic principles of Bouguet’s algorithm. The plane Π_*l*_ and plane Π_*r*_ are the image planes of Camera-1 and Camera-2 before polar correction, and the plane $\Pi_{l}^{\prime }$ and plane $\Pi_{r}^{\prime }$ are the image planes of Camera-1 and Camera-2 after polar correction. The $p_{l}^{\prime }$ and $p_{r}^{\prime }$ are the pixel coordinates of point *P* in the plane $\Pi_{l}^{\prime }$ and plane$\Pi_{r}^{\prime }$. The rotating vector *R* and translation vector *T* of the camera coordinate systems of Camera-1 and Camera-2 are obtained from camera calibration results.

In Figure 11, the practical binocular vision system can be corrected to a parallel binocular parallel system by multiplying the coordinate systems of Camera-1 and Camera-2 with their respective stereo correction matrices (*R*_*rect*_) as follows


(13) $$\left\{ \begin{array}{c} R_{l}^{\prime }=R_{rect}\cdot R_{l}\\ R_{r}^{\prime }=R_{rect}\cdot R_{r} \end{array}\right.$$

where $\{\begin{array}{c} R_{l}=R^{1/2}\\ R_{r}=R^{-1/2} \end{array}$, *R*_*rect*_ = $\left[\begin{array}{c} {\left(\frac{T}{||T||}\right)}^{T}{\left(\frac{\left[-T_{y},T_{x},0\right]}{\sqrt{T_{x}^{2}+T_{y}^{2}}}\right)}^{T}\\ {\left(\frac{T}{||T||}\times \frac{\left[-T_{y},T_{x},0\right]}{\sqrt{T_{x}^{2}+T_{y}^{2}}}\right)}^{T} \end{array}\right]$, *T* = [*T*_*x*_, *T*_*y*_, *T*_*z*_]^*T*^.

#### Shape Reconstruction

According to the morphological characteristics of spherical hedges, the surface fitting model is established by the SGBM algorithm. The SGBM algorithm is a classic semi-global matching algorithm, and this method has the advantages of both stereo matching quality and processing rates.

In the study of Romaniuk and Roszkowski (2014), the energy function of the SGBM algorithm can be represented as


(14) $$\begin{array}{c} E\left(D\right)=\sum_{P}\left(C\left(p,D_{p}\right)\right)+\sum_{q\in N_{p}}P_{1}I\left[|D_{p}-D_{q}|=1\right]\\ +\sum_{q\in N_{p}}P_{2}I\left[|D_{p}-D_{q}|>1\right] \end{array}$$

where *C*(*p*, *D*_*p*_) indicates matching cost value, *N*_*p*_ indicates pixels adjacent to point *P*, and *P*_1_ and *P*_2_ are penalty coefficient.

Considering operating efficiency, *N*_*p*_ is set to 8. The 2D search problem is divided into eight one-dimensional problems, thus using dynamic programming to treat each one-dimensional problem separately. When disparity is *d*, the matching cost value of point *P* in the *r* direction can be represented as


(15) $$\begin{array}{c} L_{r}\left(p,d\right)=C\left(p,d\right)+\min(L_{r}\left(p-r,d\right),L_{r}\left(p-r,d-1\right)\\ +P_{1},L_{r}\left(p-r,d+1\right)+P_{1},\min_{i}L_{r}\left(p-r,i\right)+P_{2})\\ -\min_{k}L_{r}\left(p-r,k\right) \end{array}$$

where *C*(*p*, *d*) is the matching cost value when disparity is equal to *d*, *min*(*L*_*r*_(*p*−*r*, *d*), *L*_*r*_(*p*−*r*, *d*−1) + *P*_1_, *L*_*r*_(*p*−*r*, *d* + 1) indicates the minimum matching cost value of previous matching point pixel of point *P* in *r* direction, and $P_{1},\min_{i}L_{r}\left(p-r,i\right)+P_{2})-\min_{k}L{}_{r}(p-r,k)$ is the constraint.

Then, the matching cost values on each path were calculated and the total sum according to the SGBM algorithm was taken. The sum of matching cost value can be expressed by


(16) $$S\left(p,d\right)=\sum_{r}L_{r}\left(p,d\right)$$

In the study of Hong and Ahn (2020), the optimal disparity *d* is corresponding to the minimum sum of matching cost value.

**FIGURE 12:**
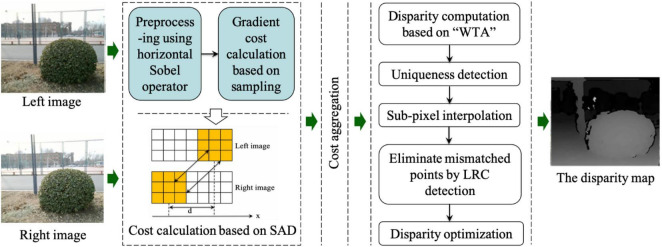
Flow chart of the improved SGBM algorithm.

This study improves the SGBM algorithm by the following two main areas: occlusion detection and disparity optimization. The left-right consistency (LRC) method is used to remove the mismatch points, and the bilateral filtering algorithm is used to fill the holes in the disparity map. Then, the corresponding point cloud coordinates of the parallax map are calculated. Figure 12 shows the flowchart of the improved SGBM algorithm.

Occlusion detection based on the LRC is used to detect the disparity of all pixels in an image. When the disparity in the left and right imaging planes is inconformity, the pixels are regarded as the occluded points. To figure out occluded points, the disparity error is defined as


(17) d(q)≠-d(q+d(q))

where *d*(*q*) is the disparity of pixel *q* in the left imaging plane (Camera-1), *d*(*q* + *d*(*q*)) is the disparity of the corresponding pixel in the right imaging plane (Camera-2) when the disparity of pixel *q* is *d*.

Disparity optimization refers to filling the holes in the disparity map. After the occlusion detection, mismatch points or occluded points are removed, and thus, some pixels have no disparity value. Meanwhile, the depth of occluded points removed by the LRC detection is greater than the depth of the object that occludes it. Therefore, the disparity of occluded points can be estimated according to the non-occluded pixels and then fill it to the disparity map. Since disparity map-based hole filling is easily led to creating stripes, an edge keeping filter is used to reduce noise and save edge information of image well. The disparity processed by bilateral filtering can be expressed as


(18) $$I_{p}^{bf}=\frac{\sum_{q\in S}G_{\sigma_{s}}\left(||\text{p-q}||\right)G_{\sigma_{r}}\left(\left|I_{p}-I_{q}\right|\right)I_{q}}{W_{p}^{bf}}$$

where, σ_*s*_ and σ_*r*_ are smooth parameters in the spatial domain and pixel range, *I*_*p*_ and *I*_*q*_ are input disparities of pixel *p* and pixel *q*, and $W_{p}^{bf}$ is the bilateral filtering weight.

The pixel coordinates of *p* and *q* are marked as *p*(*x*, *y*) and *q*(*k*, *l*), respectively. Then, *G*_σ_*s*__(||p-q||) and *G*_σ_*r*__(*I*_*p*_−*I*_*q*_) can be expressed as


(19) $$G_{\sigma_{s}}\left(||\text{p-q}||\right)=G_{\sigma_{s}}\left(x,y,k,l\right)=\exp\left(-\frac{{\left(i-k\right)}^{2}+{\left(j-l\right)}^{2}}{2\sigma_{s}^{2}}\right)$$

(20) $$G_{\sigma_{r}}\left(I_{p}-I_{q}\right)=G_{\sigma_{r}}\left(x,y,k,l\right)=\exp\left(-\frac{{||I\left(i,j\right)-I\left(k,l\right)||}^{2}}{2\sigma_{r}^{2}}\right)$$

where *I*(*i*, *j*) and *I*(*k*, *l*) are the disparity values of corresponding pixels in the disparity map.

#### Dimension Measurement of Spherical Hedges

According to the morphological characteristics of spherical hedges, the surface fitting model is established by the SGBM algorithm. The SGBM algorithm is a classic semi-global matching algorithm, which has the advantages of both stereo matching quality and processing rates.

After obtaining the disparity map through stereo matching, 3D point cloud coordinates of detected spherical hedges can be calculated by Equation 7. Then, the deformed shape of the spherical hedges is mapped and the error of coordinate and fitted coordinate of each 3D point is calculated. Finally, the coordinate of spherical hedges’ center and its radius are obtained when the sum of error is minimal.

In the calculation process, *O*(*x*_0_, *y*_0_, *y*_0_) is the center of a fitting sphere, its corresponding radius is *r*, and (*x*_*i*_, *y*_*i*_, *z*_*i*_) is the coordinate of a point cloud. The error formula of the actual coordinate and fitted coordinate of each 3D point can be expressed as (Guo et al., 2020)


(21) $$e_{i}\left(x_{0},y_{0},z_{0},r\right)={\left(x_{i}-x_{0}\right)}^{2}+{\left(y_{i}-y_{0}\right)}^{2}+{\left(z_{i}-z_{0}\right)}^{2}-r^{2}$$

Then, the sum of error is demonstrated as


(22) $$E\left(x_{0},y_{0},z_{0},r\right)=\sum_{i=1}^{N}e_{i}\left(x_{0},y_{0},z_{0},r\right)$$

where *N* is the number of 3D point clouds, and *E* is the sum of errors.

In Equation 21, *E*shows a function relation to *x*_0_, *y*_0_, *z*_0_, and *r*. Thus, all the partial derivatives with respect to *E* are set to zero, and then, a minimum value of *E* can be obtained. The extreme value of partial derivative with respect to *E* can be expressed as


(23) $$\frac{\partial E}{\partial x_{0}}=0,\frac{\partial E}{\partial y_{0}}=0,\frac{\partial E}{\partial z_{0}}=0,\frac{\partial E}{\partial r}=0$$

With Equations 20–22 can be demonstrated as


(24) $$\left\{ \begin{array}{c} \sum_{i=1}^{N}e_{i}\left(x_{i}-x_{0}\right)=0\\ \sum_{i=1}^{N}e_{i}\left(y_{i}-y_{0}\right)=0\\ \sum_{i=1}^{N}e_{i}\left(z_{i}-z_{0}\right)=0\\ \sum_{i=1}^{N}e_{i}r=0 \end{array}\right.$$

To solve out *x*_0_, *y*_0_, and *z*_0_, Equation 23 can be transformed into


(25) $$\begin{array}{c} \left[\begin{array}{ccc} \bar{x^{2}}-{\bar{x}}^{2} & \bar{xy}-\bar{x}\cdot \bar{y} & \bar{xz}-\bar{x}\cdot \bar{z}\\ \bar{xy}-\bar{x}\cdot \bar{y} & \bar{y^{2}}-{\bar{y}}^{2} & \bar{yz}-\bar{y}\cdot \bar{z}\\ \bar{xz}-\bar{x}\cdot \bar{z} & \bar{yz}-\bar{y}\cdot \bar{z} & \bar{z^{2}}-{\bar{z}}^{2} \end{array}\right]\left[\begin{array}{c} x_{0}\\ y_{0}\\ z_{0} \end{array}\right]\\ =\frac{1}{2}\left[\begin{array}{c} \left(\bar{x^{3}}-\bar{x}\cdot \bar{x^{2}}\right)+\left(\bar{xy^{2}}-\bar{x}\cdot \bar{y^{2}}\right)+\left(\bar{xz^{2}}-\bar{x}\cdot \bar{z^{2}}\right)\\ \left(\bar{x^{2}y}-\bar{x^{2}}\cdot \bar{y}\right)+\left(\bar{y^{3}}-\bar{y}\cdot \bar{y^{2}}\right)+\left(\bar{yz^{2}}-\bar{y}\cdot \bar{z^{2}}\right)\\ \left(\bar{x^{2}z}-\bar{x^{2}}\cdot \bar{z}\right)+\left(\bar{zy^{2}}-\bar{z}\cdot \bar{y^{2}}\right)+\left(\bar{z^{3}}-\bar{z}\cdot \bar{z^{2}}\right) \end{array}\right] \end{array}$$

where,


$$\bar{x}=\frac{1}{N}\sum_{i=1}^{N}x_{i},\bar{y}=\frac{1}{N}\sum_{i=1}^{N}y_{i},\bar{z}=\frac{1}{N}\sum_{i=1}^{N}z_{i},\bar{xy}=\frac{1}{N}\sum_{i=1}^{N}x_{i}y_{i},\bar{xz}=\frac{1}{N}\sum_{i=1}^{N}x_{i}z_{i},$$

$$\bar{yz}=\frac{1}{N}\sum_{i=1}^{N}y_{i}z_{i},\bar{x^{2}}=\frac{1}{N}\sum_{i=1}^{N}x_{i}^{2},\bar{y^{2}}=\frac{1}{N}\sum_{i=1}^{N}y_{i}^{2},\bar{z^{2}}=\frac{1}{N}\sum_{i=1}^{N}z_{i}^{2},\bar{x^{2}y}=\frac{1}{N}\sum_{i=1}^{N}x_{i}^{2}y_{i},$$

$$\bar{x^{2}z}=\frac{1}{N}\sum_{i=1}^{N}x_{i}^{2}z_{i},\bar{xy^{2}}=\frac{1}{N}\sum_{i=1}^{N}x_{i}y_{i}^{2},\bar{y^{2}z}=\frac{1}{N}\sum_{i=1}^{N}y_{i}^{2}z_{i},\bar{xz^{2}}=\frac{1}{N}\sum_{i=1}^{N}x_{i}z_{i}^{2},$$

$$\bar{yz^{2}}=\frac{1}{N}\sum_{i=1}^{N}y_{i}z_{i}^{2},\bar{x^{3}}=\frac{1}{N}\sum_{i=1}^{N}x_{i}^{3},\bar{y^{3}}=\frac{1}{N}\sum_{i=1}^{N}y_{i}^{3},\bar{z^{3}}=\frac{1}{N}\sum_{i=1}^{N}z_{i}^{3}.$$

Then, the radius of spherical hedges is obtained by


(26) $$\bar{x^{2}}-2x_{0}\bar{x}+x_{0}^{2}+\bar{y^{2}}-2y_{0}\bar{y}+y_{0}^{2}+\bar{z^{2}}-2z_{0}\bar{z}+z_{0}^{2}=r^{2}$$

## Results

### Binocular Vision Calibration Test and Results

**FIGURE 13:**
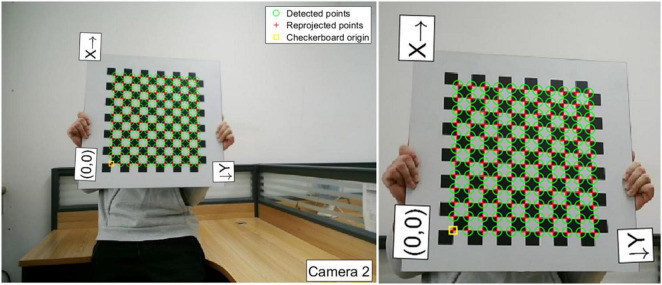
Corner extraction results.

A calibration chessboard is applied in the experiment. The chessboard is placed in front of Camera-1 and Camera-2 with different positions and attitudes, and sixteen groups of images for calibration are captured. Then, the camera calibration toolbox (Toolbox_Calib) in MATLAB is used to extract corners in the chessboard. The detailed features of the chessboard are as follows: the material is armored glass; board size is 500 mm^2^ × 500 mm^2^; chessboard size is 390 mm^2^ × 360 mm^2^; check array is 13 × 12; check size is 30 × 30 mm^2^; and the precision is ± 0.01 mm. In the captured calibration images, the number of corners that can be extracted from each image is 12 × 11. Figure 13 shows one of the corner extraction results of Camera-2.

Taking the first corner in the lower left (marked in yellow square in Figure 13 as the origin), the “X”-“Y” co-ordinate system is set up in a chessboard plane. The pixel coordinates of each corner can be obtained (Qiu and Huang, 2021). The world coordinates of corners are obtained based on the pixel coordinates of corners and check size. Then, the transformation matrix can be calculated by linear calculation. Additionally, by matrix decomposition, the intrinsic matrix (*f*_*x*_, *f*_*y*_, *u*_0_ and *v*_0_) of Camera-1 and Camera-2 can be obtained. In addition, a polynomial distortion correction model is built to correct the distortion, and the radial distortion coefficients and tangential distortion coefficients (*k*_1_, *k*_2_, *p*_1_, and *p*_2_) are given. The intrinsic parameters and distortion coefficients of Camera-1 and Camera-2 are listed in Table 1.

**TABLE 1:** The intrinsic parameters and distortion coefficients of Camera-1 and Camera-2.

No.	*f*_*x*_ (pixel)	*f*_*y*_ (pixel)	*u*_0_ (pixel)	*v*_0_ (pixel)	*k*_1_	*k*_2_	*p*_1_	*p*_2_
Camera-1	510.2063	510.0911	331.6530	243.6460	0.0678	−0.0615	0.0017	0.0004103
Camera-2	505.7195	505.9684	329.6185	248.7792	0.0884	−0.0980	0.002834	0.0007345

**FIGURE 14:**
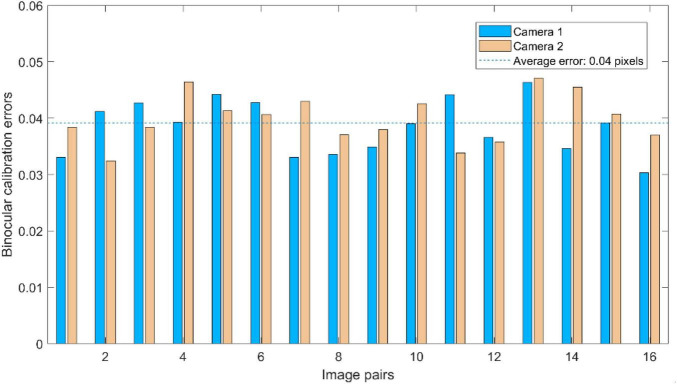
Binocular calibration errors of each image pairs.

To test the calibration accuracy results listed in Table 1, the calibration errors of captured calibration images are analyzed, respectively. The coordinates of the corners in the “X”-“Y” co-ordinate system are obtained after back-projection and compared with the corresponding actual pixels of corners in the chessboard to obtain calibration errors. The binocular calibration errors of each image pair are shown in Figure 14. As can be seen in Figure 14, the binocular calibration errors for each pair of images are less than 0.05 pixels, and the average errors of Camera-1 and Camera-2 are both 0.04 pixels.

**FIGURE 15:**
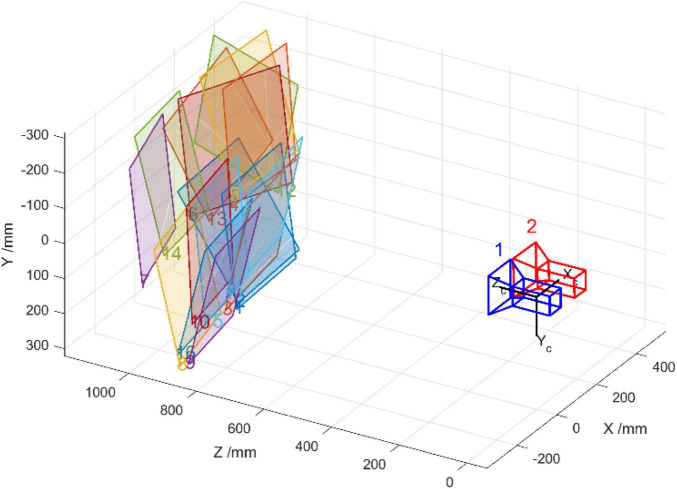
The position and attitude relationship between cameras and calibration chessboard.

Then, the binocular vision calibration proceeds by using the binocular calibration toolbox in MATLAB. The installation of the two cameras is close to the coplanar and row alignment. As shown in Figure 15, the “1” and “2” represent the position and placing attitude of Camera-1 and Camera-2, respectively. The sixteen colored squares represent the positions and placing attitudes of the sixteen images of the calibration chessboard. In addition, the relative position between Camera-1 and Camera-2 can be obtained. Iterate over the intrinsic parameters and distortion coefficients of Camera-1 and Camera-2 obtained by monocular vision calibration. The transformation matrix and vector between Camera-1 and Camera-2 are given as *T* = −119.2486 0.3206 3.3474]^*T*^ and $R_{rect}=\left[\begin{array}{c} {\left(\frac{T}{\left\|T\right\|}\right)}^{T}{\left(\frac{\left[-T_{y},T_{x},0\right]}{\sqrt{T_{x}^{2}+T_{y}^{2}}}\right)}^{T}\\ {\left(\frac{T}{\left\|T\right\|}\times \frac{\left[-T_{y},T_{x},0\right]}{\sqrt{T_{x}^{2}+T_{y}^{2}}}\right)}^{T} \end{array}\right]$.

The binocular calibration errors are also obtained by reverse projection of spatial coordinates of the corners, the binocular calibration errors for each pair of images are less than 0.07 pixels, and the average error of binocular calibration is less than 0.04 pixels. The calibration accuracy meets the requirements of the binocular vision system in this study.

**FIGURE 16:**
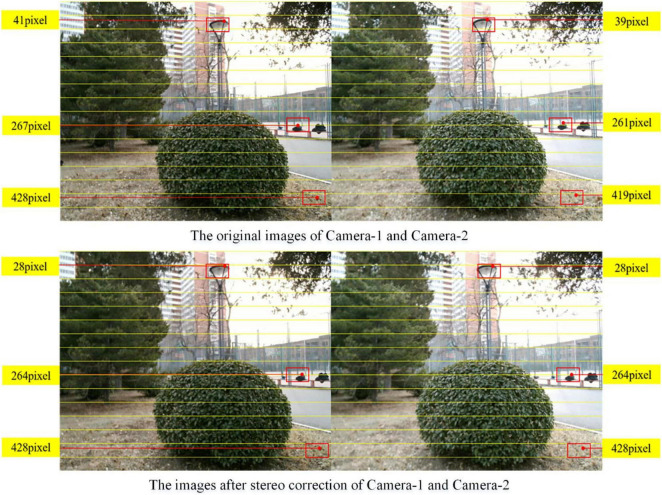
Comparison of before and after stereo correction.

Afterward, the images collected by this binocular vision system outdoor are used for stereo correcting, and the result is shown in Figure 16. The pixels of red dots from the top of the image are marked on the images. The pixels from the top of the original image of Camera-1 are 41, 267, and 428, whereas the values of Camera-2 are 39, 261, and 419, respectively. Herein, after stereo correction, the pixels of the same object in images of Camera-1 and Camera-2 are in the same row, and the pixels of makers after stereo correction are all 28, 264, and 428, respectively.

### Laboratory Test and Results

**FIGURE 17:**
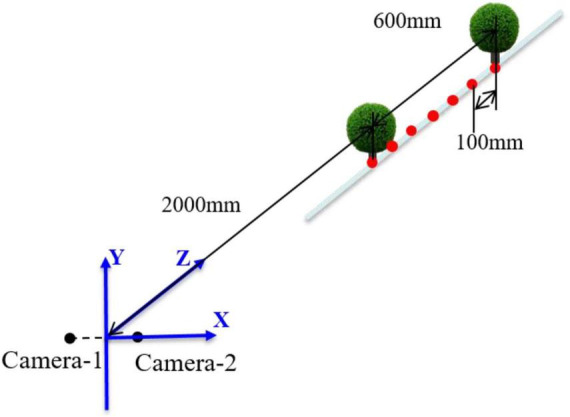
Schematic diagram of laboratory test.

To better reflect the 3D reconstruction effect of spherical hedges, a standard spherical hedge with a diameter of 60 mm was used to conduct a laboratory test first. The different test data sets could be obtained by changing the distances between the spherical hedges and the binocular vision system. Then, the stability and accuracy of this measurement system were verified according to the errors of the measured value and actual value. In the laboratory test, a straight line was marked in front of the binocular vision system, and seven different positions were set at the direction of Z by every 100 mm in range of 2,000–2,600 mm, described as red dots in Figure 17. Seven groups of images were captured, and the test values of the spherical center and its radius are shown in Table 2.

**TABLE 2:** Laboratory test results of center coordinate and its radius.

No.	AR (mm)	MR (mm)	AER (mm)	RER (%)	AL (mm)	ML (mm)	LD (mm)
1	300	301.10	1.10	0.37	(100, −300, 2,000)	(101.54, −299.03, 2,018.79)	18.88
2	300	301.24	1.24	0.41	(100, −300, 2,100)	(102.17, −302.5, 2,111.72)	12.17
3	300	301.71	1.71	0.57	(100, −300, 2,200)	(103.3, −303.7, 2,211.05)	12.03
4	300	302.24	2.24	0.75	(100, −300, 2,300)	(103.25, −305.21, 2,318.11)	19.12
5	300	300.67	0.67	0.22	(100, −300, 2,400)	(104.03, −305.67, 2,414.41)	16.00
6	300	301.08	1.08	0.36	(100, −300, 2,500)	(104.36, −306.59, 2,514.02)	16.10
7	300	297.00	3.00	1.00	(100, −300, 2,600)	(105.27, −308.54, 2,613.91)	17.15

*AR, actual radius; MR, measured radius; AER, absolute error of radius; RER, relative error of radius; AL, actual location; ML, measured location; LD, location deviation. The average absolute error of radius is 1.58 mm. The average relative error of radius is 0.52%. The RMSE of radius is 1.59 mm. The average location deviation is 15.92 mm. The RMSE of location deviation is 2.66 mm.*

According to Table 2, the maximum and average error of radius of standard spherical hedges by the proposed system were 3.00 mm and 1.58 mm, respectively; maximum and average relative errors of radius were 1.00% and 0.52%, respectively; the root mean square error (RMSE) of the radius was 1.59 mm. Moreover, the relative error and error of radius increase with the distance in direction of Z, and the maximum relative error was 1.00% at the distance of 2,600 mm in direction of Z, which indicated the high monitoring accuracy and stability of the proposed system for radius measurement. The minimum, maximum, and average location deviations were 12.03, 19.12, and 15.92 mm in the range of 2,000–2,600 mm, and the RMSE of the center coordinate of spherical hedges was 2.66 mm. It showed that the proposed system had high accuracy in positioning and dimension measurement and had stability and applicability for different distances in a certain range.

### Outdoor Test and Results

**FIGURE 18:**
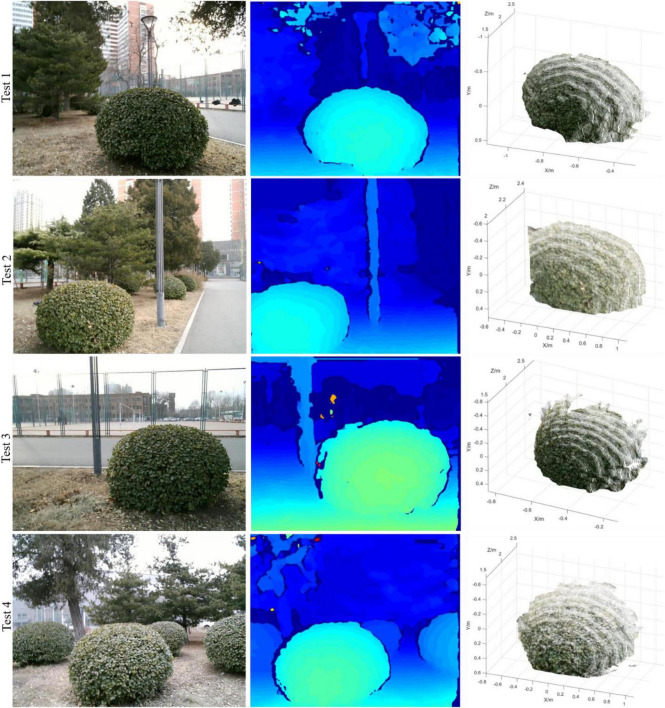
3D reconstruction result of outdoor test.

An outdoor test was conducted at China Agricultural University East Campus (Beijing, China). During the test, the weather was overcast and the leaves of spherical hedges were slightly yellow and sparse. A number of four spherical hedges were randomly selected on the campus; therefore, the results have a certain generality. The spherical hedges were non-standard spheres and their radius was unknown; therefore, for each spherical hedge, six groups of images were captured at different positions. The distances between the proposed system and spherical hedges were all around 2,000 mm. The outdoor scene image acquired by the left camera, the disparity map obtained by stereo matching, and the 3D shape reconstruction image of the proposed system are shown in Figure 18.

In the outdoor test, the actual center position and radius of spherical hedges were measured manually using a tap. In each test, the actual radius was collected manually by six different positions, and the average value was determined. The results of the center coordinate and its radius in Figure 18 are shown in Table 3.

**TABLE 3:** Outdoor test results of center coordinate and its radius.

No.	AR (mm)	MR (mm)	AER (mm)	RER (%)	AL (mm)	ML (mm)	LD (mm)
1	962.42	957.28	−5.14	0.53	(50, −80, 2,000)	(41.36, −67.50, 1,988.91)	18.84
2	881.92	877.89	−4.03	0.46	(150, −20, 2,150)	(152.35, −18.37, 2,165.26)	15.53
3	887.25	884.85	−2.4	0.27	(110, −150, 2,000)	(108.24, −145.38, 2,018.63)	19.27
4	932.75	937.26	4.51	0.48	(120, −80, 2,050)	(116.88, −73.21, 2,168.02)	19.51

*AR, actual radius; MR, measured radius; AER, absolute error of radius; RER, relative error of radius; AL, actual location; ML, measured location; LD, location deviation. The average absolute error of radius is 4.02 mm. The average relative error of radius is 0.44%. The RMSE of radius is 1.01 mm. The average location deviation is 18.29 mm. The RMSE of location deviation is 1.61 mm.*

According to Table 3, the maximum and average errors of the radius of measured spherical hedges in the outdoor test were 5.14 and 4.02 mm, respectively; maximum and average relative errors of radius were 0.53% and 0.44%; the and RMSE of the radius was 1.01 mm, respectively. At the distance of around 2,000 mm in direction of Z, the maximum and average location deviation were 19.51 and 18.29 mm, respectively. It indicated a high measurement accuracy and stability of the proposed system for outdoor sphere center positioning and radius detection.

## Discussion

A binocular vision system for spherical hedge reconstruction and measurement was proposed in this work to provide front-end visual information for pruning robots. Through theoretical analysis and experimental verification, this shape reconstruction and dimension measurement method showed high accuracy in both spherical center positioning and radius measurement. The conclusions of this study were as follows:

(1) The binocular vision platform was built based on the theory of binocular parallel structure. After binocular camera calibration, stereo image correcting was used based on Bouguet’s algorithm to improve the accuracy of shape reconstruction. Meanwhile, the captured 2D images were processed through filtering algorithm, segmentation, edge extraction, etc. Then, an improved SGBM algorithm was applied to obtain a good disparity map.

(2) The sharp reconstruction and measurement method were tested in a laboratory and outdoors in the detection range of 2,000–2,600 mm. The laboratory test result showed that the average error and average relative error of standard spherical hedges radius were 1.58 mm and 0.53%, respectively; the average location deviation of the center coordinate of spherical hedges was 15.92 mm in range of 2,000–2,600 mm. The outdoor test showed that the average error and average relative error of spherical hedges radius by the proposed system were 4.02 mm and 0.44%, respectively; the average location deviation of the center coordinate of spherical hedges was 18.29 mm. Therefore, the proposed system could be employed for the visual information acquisition of various trimming robots due to its excellent applicability.

Future studies may involve expanded tests on different shapes of hedges to clarify the accuracy and stability of the proposed system further. This study provides key technical support for visual detection in studies of trimming robots.

## Data Availability Statement

The original contributions presented in the study are included in the article/supplementary material, further inquiries can be directed to the corresponding author/s.

## Author Contributions

YZ, JG, TR, and HL built the system, conducted the experiments, and wrote the manuscript. BZ, JZ, and YY designed the measurement method. All authors discussed the measurement method and designed the laboratory and outdoor experiments.

## Conflict of Interest

The authors declare that the research was conducted in the absence of any commercial or financial relationships that could be construed as a potential conflict of interest.

## Publisher’s Note

All claims expressed in this article are solely those of the authors and do not necessarily represent those of their affiliated organizations, or those of the publisher, the editors and the reviewers. Any product that may be evaluated in this article, or claim that may be made by its manufacturer, is not guaranteed or endorsed by the publisher.
